# Acute Coronary Syndrome or Right Atrial Cardiac Myxoma? An Atypical Presentation

**DOI:** 10.7759/cureus.19116

**Published:** 2021-10-29

**Authors:** Pejmahn Eftekharzadeh, Shahzad Ahmed

**Affiliations:** 1 Internal Medicine, Lower Bucks Hospital, Bristol, USA; 2 Cardiology, Lower Bucks Hospital, Bristol, USA

**Keywords:** acute coronary syndrome, intracardiac thrombus, primary cardiac tumors, left atrial myxoma, right atrial myxoma

## Abstract

The size and location of cardiac tumors determine how patients present with signs of heart failure due to diminished cardiac output within the circulatory system. Poor cardiac output presents with signs of heart failure, which include pulmonary edema, lower extremity edema, jugular venous distention, dyspnea, orthopnea and can be insidious in onset. Vital signs on presentation can often be abnormal and patients may present hemodynamically unstable. We present a case of a female who presented to the emergency room after experiencing a sudden onset of substernal, pressure-like chest pain while sleeping. Vital signs on presentation were stable with no evidence of heart failure symptoms as listed above. Cardiac catheterization showed patent coronary arteries but was found to have a 5.8 x 4.7 x 3.5 cm hypervascular cardiac myxoma located in the right atrium. Instead of a typical heart failure presentation, as any space-occupying mass would decrease the effective cardiac output, the patient presented with angina. During the procedure, the mass was noted to be perfused by the left circumflex artery, creating coronary steal phenomenon, shifting blood away from the coronary arteries and into the mass, causing ischemic anginal pain. The patient ultimately underwent surgical excision of the lesion and her anginal symptoms resolved.

## Introduction

The incidence of primary cardiac tumors is rare. The most common cardiac tumors are atrial myxomas with left-sided tumor predominance [1]. Other cardiac masses such as thrombi or vegetations are more frequently identified. Due to the rare incidence of primary cardiac tumors and their nonspecific presentation, the evaluation and diagnosis are often challenging. In this case report, we discuss a patient who presented with symptoms similar to those of acute coronary syndrome. However, on further evaluation, the patient was found to have atrial myxoma.

## Case presentation

A 61-year-old Asian female with a past medical history of diabetes mellitus type 2, dyslipidemia, hypertension, and gastroesophageal reflux disease (GERD) presented to the Emergency Department (ED) with a complaint of chest pain that woke her up at 2 A.M. The pain was burning in nature, located in the epigastric region, and radiated to her back. Given her history of GERD, the patient took esomeprazole followed by ranitidine. Both provided only minimal relief. Five hours later, the patient developed intermittent squeezing, pressure-like left-sided chest pain under the left breast. In the ED, the chest pain was relieved by nitroglycerin paste. On review of systems, the patient denied any associated palpitations, dyspnea, diaphoresis, or nausea. The patient confirmed no cardiac disease in her past medical history and she denied any family history of cardiac disease. 

**Figure 1:**
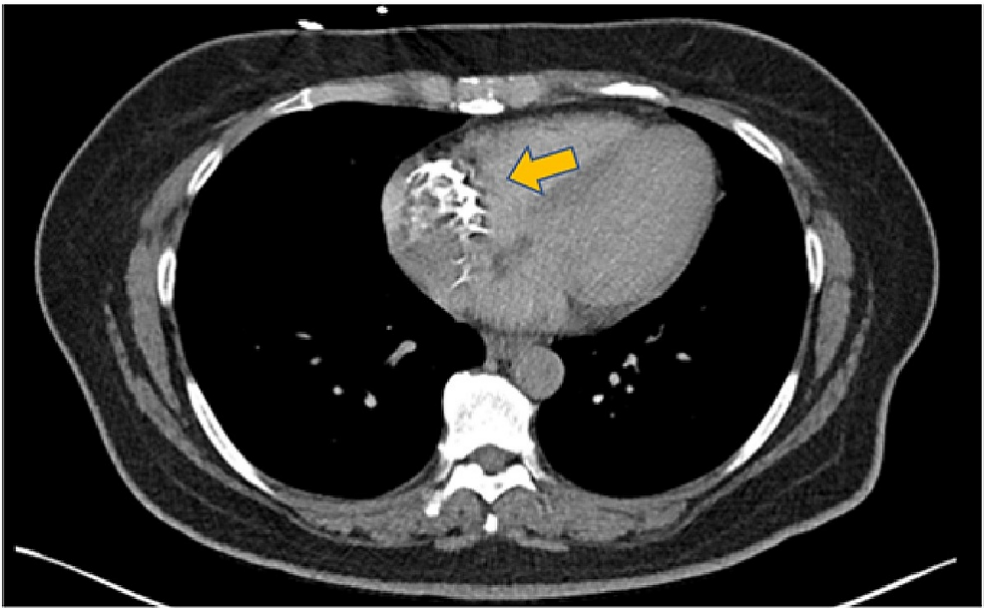
CT scan of chest, abdomen, and pelvis with IV contrast demonstrating a 4.5 to 5.0cm (gold arrow) area of calcification located in the right atrium

At the time of presentation, the patient’s vitals were stable. Physical exam showed a patient who was in obvious pain demonstrated by Levine’s sign, but cardiac exam showed a regular rate and rhythm, no murmurs, gallops, and normal first heart sound (s1), and second heart sound (s2). The respiratory exam showed lungs clear to auscultation bilaterally without any wheezes, rales, or rhonchi. There was neither jugular venous distention nor any lower extremity edema. An ECG was done, showing normal sinus rhythm with a heart rate of 61 beats per minute (BPM), without ST elevation or depression. A computed tomography (CT) scan of the chest, abdomen, and pelvis with IV contrast showed a 4.5 to 5.0 cm area of calcification in the right atrium as noted in Figure 1. 

**Figure 2:**
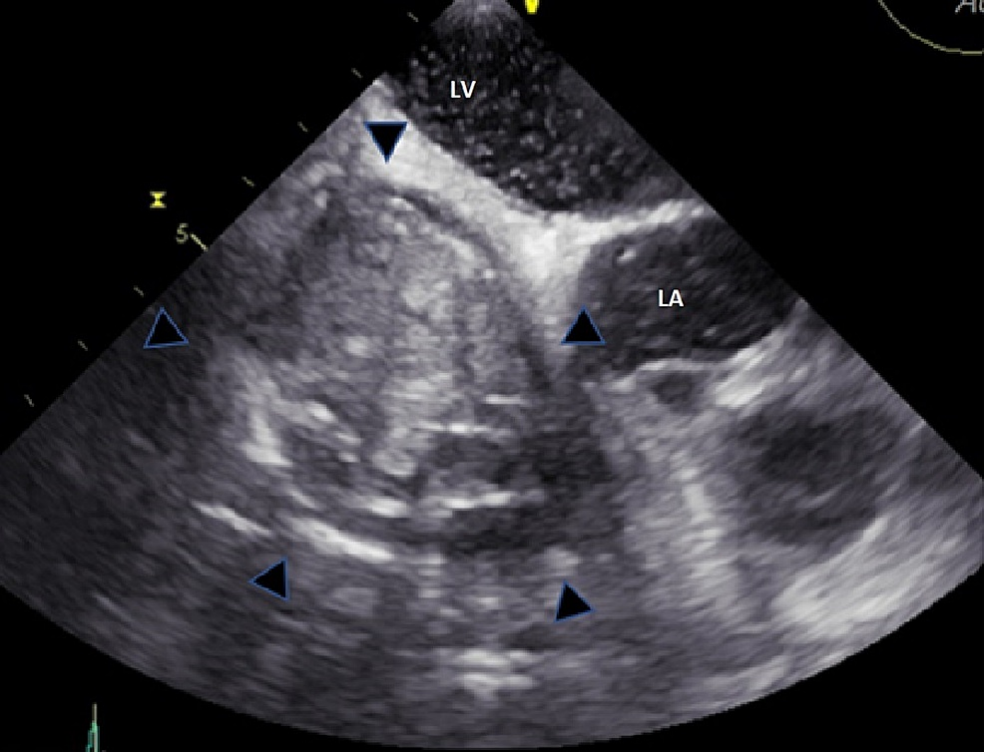
Large oval-shaped heterogeneous intracardiac mass (encircled by black wedges) measuring 3.3 x 4.2 cm in the right atrium LA: Left atrium, LV: Left ventricle

Given this finding of an unknown intracardiac mass, a transesophageal echocardiogram (TEE) with color Doppler flow was performed to further evaluate the possibility of the lesion being an intracardiac mass or thrombus. The study showed a large oval-shaped heterogeneous intracardiac mass measuring 3.3 x 4.2 cm in the right atrium. This tumor did not obstruct the blood flow of the tricuspid valve nor did it impact the venous return from the inferior vena cava as demonstrated in Figure 2.

**Figure 3:**
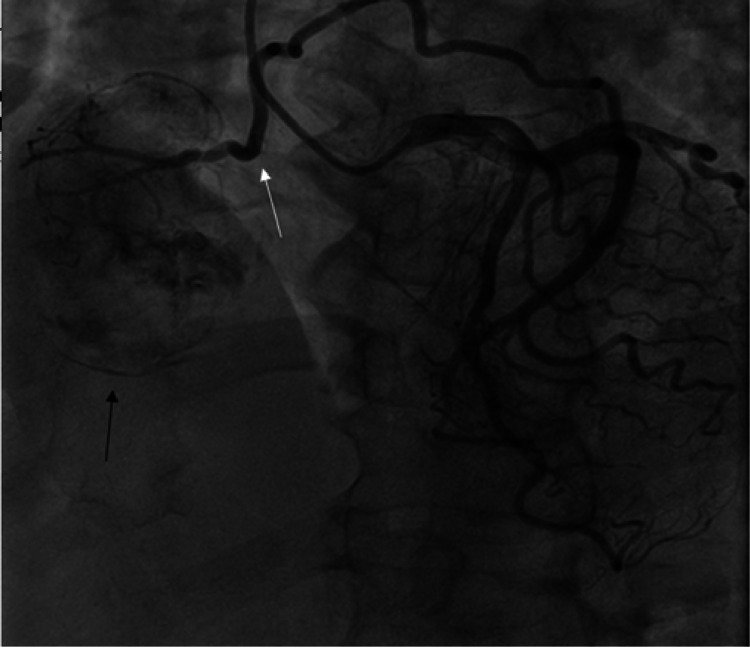
Left circumflex artery (white arrow) perfusing a right atrial mass (black arrow)

Cardiac catheterization was performed to serve two purposes i.e. to rule out ischemia given the patient’s symptoms as well as risk stratification for her preoperative evaluation as she would need surgical excision of the lesion. This study showed patent coronary vessels with a large hypervascular mass being supplied by the left circumflex artery (LCx) in the right atrium. The image of the cardiac catheterization is depicted in Figure 3.

**Figure 4:**
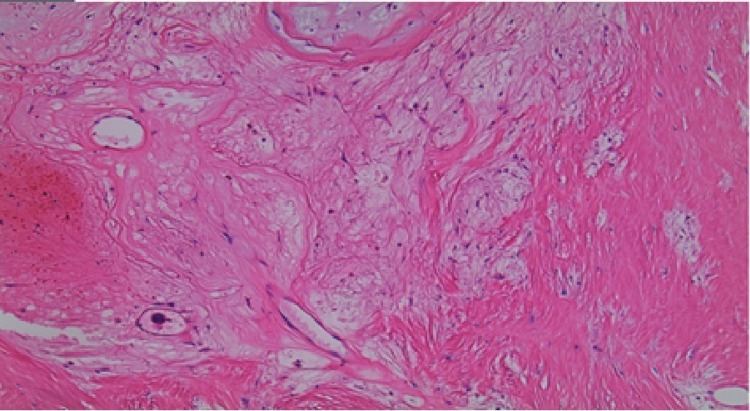
Pathology image of the right atrial myxoma consistent with hemorrhage, fibrosis, calcification and focal ossification

Cardiopulmonary bypass and excision of the right atrial mass were performed by a cardiothoracic surgeon at a tertiary care center. The specimen was sent to pathology where it was determined to be a myxoma with hemorrhage, fibrosis, calcification, and focal ossification, totaling 5.8 x 4.7 x 3.5 cm^3^ in size. The histology is illustrated in Figure 4.

After surgical excision, the patient remained symptom-free without any anginal symptoms. The patient did experience a postoperative fever but her white blood count was within normal limits and blood cultures at 48 hours were negative. The patient was discharged on postoperative day two in stable condition. 

## Discussion

This patient’s presentation was atypical for that of an atrial myxoma. Normal right-sided cardiac chambers rely on a tricuspid valve area of 5 m^2^ to allow efficient forward flow circulation. Interestingly, this large mass (5.8 x 4.7 x 3.5 cm^3^) did not show evidence of obstruction in circulation and the patient presented hemodynamically stable with normal vital signs. Next, we consider the space this mass occupies relative to the right atrium. According to Karki et al., the normal volume of blood a right atrium can hold at one time is between 11 to 40 mLs [2]. We calculated the estimated volume of this ellipsoid mass using the volume formula V=4/3π(x)(y)(z), where (x), (y), & (z) represent the radius, in cm, of each three dimensions of the mass. The volume we estimate the patient’s lesion to take is ~50cm^3^ of blood, which is greater than the entire size of the right atrium in most individuals. Because of this discrepancy, we can infer some right atrial enlargement in this patient’s cardiac chamber size. Furthermore, given the space-occupying lesion in a right atrium, we would expect physical exam findings similar to those of right heart failure due to the presumed lack of forward blood flow from an effectively “smaller” chamber space. Such signs include lower extremity edema, pulmonary edema, and prominent “a waves” in the jugular veins. None of these findings were present in our patient's physical exam. We also could have expected to hear either an early diastolic rumble (due to obstruction of the tricuspid valve) or a holosystolic murmur (due to tricuspid regurgitation) [3], but then again our patient did not have either of these findings.

A comprehensive literature review was performed on typical presentations of cardiac myxomas, which are detailed below. These typical presentations are not consistent with our case subject with chest pain but shed light on the pathogenesis of right atrial myxomas and the restriction of forward blood flow from the right side of the heart. In one typical case, a 51-year-old male with a recent diagnosis of heart failure (New York Heart Association Class III) presented with fatigue and dyspnea. He had mild dyspnea for two years and had developed a three-month history of nonproductive cough and night sweats. On examination, the patient was found to have mild central cyanosis and a mid-diastolic murmur with an occasional systolic click at the mitral and tricuspid areas. Echocardiography displayed a 7 cm mass occupying almost the entire right atrium. The mass was on a long stalk, prolapsing through the tricuspid valve. The patient did not display any anginal symptoms but did present insidiously with circulatory symptoms of dyspnea, cyanosis, and a heart murmur [4]. In another case, Saadeh et al. present a 38-year-old female with dyspnea and recurrent syncopal episodes. Upon investigation, the patient had evidence of right-sided heart failure with associated tricuspid regurgitation and pulmonary hypertension. A mass was located which originated below the fossa ovalis affecting the tricuspid valve causing a right ventricular outflow obstruction [5]. Both these cases in our literature review portrayed a right atrial myxoma which caused valvular dysfunction, thus leading to a right ventricular outflow tract obstruction resulting in their ultimate presentation. Our patient did not have valvular involvement, so understandably her hemodynamic status was preserved in our case.

One case report of an atrial myxoma did present with chest pain similar to our case. Setty et al.’s case of a 38-year-old female with a history of intermittent episodes of presyncope presented with chest pain, epigastric abdominal pain, and emesis for three days. The cardiovascular examination did not show any clinically detectable murmurs, similar to our case. A transesophageal echocardiogram confirmed the presence of a 4 cm right atrial mass attached to the interatrial septum that was prolapsing through the tricuspid valve into the right ventricle [6]. In our case, the mass was slightly larger, but with a similar location and a similar presentation with chest pain.

In our case, cardiac catheterization determined that the blood supplying the patient’s mass originated as a fistula from the LCx artery causing coronary steal phenomenon. Coronary steal phenomenon refers to myocardial ischemia caused by the diversion of blood away from normal myocardial circulation [7]. In this case, the LCx artery will have difficulty supplying blood to the cardiac tissue as the mass is "stealing" blood from the LCx artery. As a result, this will decrease blood flow to the coronary arteries supplying cardiac tissue and this "ischemia" leads to the patient's pain.

Next, we discuss her response to nitroglycerin. After the medication was administered, her pain improved. We must consider the likelihood of esophageal hyper-contraction causing her symptoms as these spasms can mimic angina. Since nitroglycerin relaxes the smooth muscle in the esophagus and can cause vasodilation along the vascular bed, it may be difficult to differentiate myocardial ischemia from esophageal hyper-contraction. According to Herregods et al., esophageal hyper-contraction presents with dysphagia to both solids and liquids in roughly 67% of patients diagnosed with this disorder [8]. As she did not experience any dysphagia, we can infer that nitroglycerin helped with vasodilatory effects to augment blood flow to cardiac tissue to alleviate her symptoms as opposed to relaxing the smooth muscle of the esophagus. In addition, the patient's anginal symptoms completely resolved after the mass was removed making a coronary steal phenomenon more likely and esophageal hypercontraction less likely.

The timing of presentation and its relationship to the rate of growth in myxomas is another interesting point to address. While the average growth rate of right atrial myxomas is unknown, in one case report the right atrial myxomas had a growth rate of 1.36 × 0.3 cm/month. This indicates that cardiac masses may have been present for many months, or years, and may have gone undetected due to lack of symptoms [9]. Unfortunately, the patient had never had a prior ECG or CT scan to determine the growth rate of her myxoma.

## Conclusions

Primary cardiac tumors remain rare and challenging to diagnose because the presenting symptoms vary greatly. Depending on their size and location in the atrium, they may present with hemodynamic instability due to flow obstruction. Our patient with a relatively large cardiac myxoma was hemodynamically stable with an absence of right-sided heart failure symptoms such as dyspnea, jugular venous distention, cardiac murmurs, or lower extremity edema. Instead, our patient presented with angina attributed to coronary steal phenomenon which diverted blood away from the coronary vessels and into the mass, causing ischemia to the myocardium. After our patient underwent surgical excision of her cardiac myxoma her anginal symptoms resolved entirely.
